# Nutrients recovery from dairy wastewater by *Chlorella vulgaris* and comparison of the lipid’s composition with various chlorella strains for biodiesel production

**DOI:** 10.1371/journal.pone.0297464

**Published:** 2024-04-10

**Authors:** Nikita Zibarev, Amira Toumi, Natalia Politaeva, Igor Iljin

**Affiliations:** 1 Laboratory "Interdisciplinary Research and Education on Technological and Economic Problems of Energy Transition (CIRETEC-GT)", Peter the Great St. Petersburg Polytechnic University, Saint Petersburg, Russia; 2 Graduate School of Biotechnology and Food Science, Peter the Great St. Petersburg Polytechnic University, Saint Petersburg, Russia; Hainan University, CHINA

## Abstract

Microalgae biomass is regarded as a promising feedstock for biodiesel production. The biomass lipid content and fatty acids composition are among the main selective criteria when screening microalgae strains for biodiesel production. In this study, three strains of *Chlorella* microalgae (*C*. *kessleri*, *C*. *sorokiniana*, *C*. *vulgaris*) were cultivated nutrient media with different nitrogen contents, and on a medium with the addition of dairy wastewater. Moreover, microalgae grown on dairy wastewater allowed the removal of azote and phosphorous. The removal efficiency of 90%, 53% and 95% of ammonium nitrogen, total nitrogen and phosphate ions, respectively, were reached. The efficiency of wastewater treatment from inorganic carbon was 55%, while the maximum growth of biomass was achieved. All four samples of microalgae had a similar fatty acid profile. Palmitic acid (C16:0) was the most abundant saturated fatty acid (SFA), and is suitable for the production of biodiesel. The main unsaturated fatty acids (UFA) present in the samples were oleic acid (C18:1 n9); linoleic acid (C18:2 n6) and alpha-linolenic acid (C18:3 n3), which belong to omega-9, omega-6, omega-3, respectively.

## Introduction

Photosynthetic microalgae are promising sources of various types of renewable biofuels; alternative to traditional energy crops [1,2]. Microalgae are used to produce fuels such as methane during anaerobic digestion, biohydrogen, and biodiesel [3–9]. The production of biodiesel and omega-3 from microalgae lipids are among the most developing areas of application. Indeed, under optimal cultivation conditions, certain types of microalgae can cumulate more lipids than oleaginous plants. For instance, the lipid content of: *Scenedesmus dimorphus* is comprised between 16–40%, *Prymnesium parvum*– 22–38%, *Euglena gracilis*– 14–20%, *Chlorella vulgaris*– 14–22%, *Dunaliella salina*– 16–44%, *Haematococcus pluvialis*– 25–45%, *Tetraselmis suecica*– 20–30%, *Isochrisis galbana*—22–38%, *Nannochloropsis sp*.– 33–38%, *Stichococcus sp*.– 40–59%, and *Botryococcus braunii*–up to 80% [10,11].

The extraction of lipids from microalgae biomass is a crucial step of the biodiesel production process. Furthermore, it is also crucial to select productive microalgae strains, suitable for this particular application. The genus *Chlorella* includes species that produce varying amounts of lipids. For example, *Chlorella vulgaris* accumulate from 14% to 22% of lipids, *Chlorella pyrenoidosa* from 2% to 12% [12]. One of the hindering factors of the development of microalgae biofuels industry is the cost of cultivation and biomass processing. A promising approach for reducing the costs related to microalgae cultivation is the use of wastewater from food processing plants as a nutrient medium. For instance, the composition of wastewater from the dairy industry consists of biogenic elements including nitrogen and phosphorus, which are necessary for the growth of microalgae [13,14]. Therefore, such a wastewater could be used as a basis for creating a nutrient medium for microalgae cultivation. Such a combination will not only generate biomass, but also purify wastewater from pollutants [15,16]. Studies have been carried on the use of microalgae for the treatment of wastewater from various industrial sectors such as the textile, pharmaceutical, dairy, and alcoholic drinks [17–20]. High purification efficiency has been demonstrated by microalgae of the genus *Chlorella* (hereinafter *C*.). It has been shown that such species as *C*. *vulgaris*, *C*. *sorokiniana*, *C*. *kessleri* are able to purify wastewater from medicinal compounds (ibuprofen, diclofenac, carbamazepine, levofloxacin) by up to 100%, as well as to reduce concentrations of nitrogen, phosphorus, COD compounds by 75–95%, while accumulating biomass at a rate of 0.64–14.8 g/l/day with a lipid content of 39–42% [21,22]. In previous papers, *C*. *kessleri* grown in brewery wastewater was shown to effectively remove pollutants and accumulate biomass. Moreover, when added to food waste for anaerobic digestion for biogas production, the biomass allowed to obtain higher yields of methane [23,24].

The objective of this work is the study of the nutrient recovery from dairy wastewater by *Chlorella vulgaris*, as well as the comparison of the lipid content and fatty acid composition of three *Chlorella* species cultivated on various media for assessing their potential use for biodiesel production.

## Materials and methods

### Microalgae strains

Four samples of dry *Chlorella* biomass samples were studied in order to select the most suitable strain for the production of lipids:

- Sample N°1: *C*. *kessleri* strain VKPM A1-11 ARM [25] obtained in a dry form directly from the Scientific and Production Association (SPA) "Algobiotechnology", Novovoronezh, Russia. There is no data on the growth rate of this sample under growing conditions applied by the producer;
- Sample N°2: *C*. *kessleri* strain VKPM A1-11 ARM, cultivated in the laboratory "Industrial Ecology" SPbPU, St. Petersburg, Russia;
- Sample N°3: suspension of *C*. *vulgaris* strain GKO VKPM Al-24 [26], obtained from the company "Algotek", Tver, Russia;
- Sample N°4: *C*. *sorokiniana* microalgae strain 211-8k, obtained from the algae collection of the University of Göttingen (SAG), Göttingen, Germany.

### Cultivation conditions

In this study, samples N°2 and N°4 were grown on a modified Hoagland solution as described in the literature [27,28]. One of the most important nutrients affecting the growth of microalgae is nitrogen. It is known that nitrogen deficiency contributes to the accumulation of neutral lipids [29], therefore, the concentration of this compound in the nutrient medium was varied. Based on the results of a previous study [29], the nutrient medium for sample N°2 was prepared with a concentration of 0.15 g/l KNO_3_.

Sample N°1 was cultivated by the manufacturer and the biomass was obtained directly in a freeze-dried form.

For the cultivation of sample N°3, a concentrated suspension of microalgae with an optical density ОD_750_ = 1.61 was first diluted with the nutrient medium to an optical density ОD_750_ = 0.45. After that, the diluted microalgae *C*. *vulgaris* suspensions in their initial nutrient medium were mixed with the dairy wastewater of the Wimm-Bill-Dann LLC dairy plant (Russia, St. Petersburg) at a ratio of 30:70 (effluent water to *C*. *vulgaris* suspension). According to Table 1, untreated Wimm-Bill-Dann LLC wastewater contains nitrogen and phosphorus compounds, which are essential macronutrients for the growth of microalgae [30]. The used wastewater was obtained after passing through the stage of flotation treatment and added to the microalgae on the day of sampling.

**Table 1:** Wimm-Bill-Dann wastewater composition.

pH	COD	Total N	NH^4+^	NO^2−^	NO^3−^	Total Р	PO^4−^	BOD_5_
-	mg/l	mg/l	mg/l	mg/l	mg/l	mg/l	mg/l	mg/l
9.94	5650	47	2	0.15	6	20	14.20	2060

Samples N°2, N°3 and N°4 were grown in photobioreactors described in our previous study *(see PBR-3)* [31]. Microalgae were washed three times with distilled water, and then added to 50 liters of nutrient medium till obtaining an initial cell concentration of 11 million cells/ml. The cultivation of microalgae was carried out for 8 days. A constant water temperature was maintained at 28 ± 2°C using a thermostat. The suspensions were bubbled with atmospheric air at a rate of 1.5 l/min under continuous illumination with LED light panel at an intensity of 135 micromole photons m^-2⋅^s^-1^. The growth of microalgae was measured based on the optical density at 750 nm and by cell counting on a Goryaev chamber.

### Obtaining dry biomass

Microalgae biomass was harvested by centrifugation for 20 minutes at 4600 rpm using a 12-liter Thermo scientific Sorvall RC 12BP+ centrifuge. The pellets were then frozen at -24°C for 18h. After that, the biomass was lyophilized using the AK5-50Н freeze dryer (Proflab) using a three-stage process (stage 1: 0.5 mbar; 50°C, 24 h → stage 2: 0.5 mbar, 50°C for 24 hours→ stage 3: 0.3 mbar, 50°C, 3 h). The moisture content of the dry biomass was determined by gravimetric analysis. For that, the lyophilized biomass samples were placed in pre-dried weighing bottles and dried in a laboratory drying oven SNOL 200/200 at 105°C, for about 120 min till reaching constant weight. The moisture content (W, wt. %) is calculated by the Formula 1:

$$W=[(m_{1}‐m_{2})\cdot 100]:\;(m_{1}‐m_{0})$$ (1)

where m_0_ is the mass of the pre-dried weighing bottles, g; m_1_ is the mass of the weighing bottle containing the biomass sample prior drying, g; m_2_ is the mass of the weighing bottle with the biomass sample after drying, g.

### Lipids extraction

Each sample of dry biomass (3 g) was first disintegrated by ultrahomogenization on ice at 10 000 rpm for 5 minutes using the Silent Crusher M homogenizer. After that, the samples were subjected to Soxhlet extraction using a solvent extraction mixture of hexane:ethanol at a ratio of 9:1. The extraction step took place for 130 min (15 cycles) at 100°C followed by a 30 minute rinsing step at 100°C and a drying step at a temperature of 150°C. The drying time was determined visually by the degree of evaporation of the solvent. After that, the extracts were dried at 55°C to constant mass in a laboratory oven. The experiments were performed in triplicate.

### Assessment of the removal efficiency of nutrients by microalgae

The assessment of the removal efficiency of nutrients was identified measuring several indicators: pH, total nitrogen (TN), phosphate ions (PO_4_^3-^), ammonium ions (NH_4_^+^), inorganic carbon (IC). For that, the suspensions were centrifuged for 10 minutes at a rotation speed of 2500 rpm and the supernatants were recovered and diluted 20 times. The analyzer L CPN, (SHIMADZU) was used to determine the TN and IC. The content of ammonium ions was determined according by Nessler method. The concentration of phosphate ions was determined by photometric method at 750 nm, based on the reduction of ammonium molybdate in an acid solution. The pH value was measured using the I-160MI ionomer. The removal efficiency is calculated by the Formula 2:

$$E=((C_{1}‐C_{2}):C_{1})\cdot 100\%$$ (2)

where С_1_ –is the initial concentration of the nutrient in wastewater, mg/l;

С_2_ –is the final concentration of the nutrient, after purification by microalgae, mg/l.

### Determination of the fatty acid composition of the obtained lipids

The fatty acid composition of lipids obtained was determined by gas chromatography. The analysis was carried out on a gas chromatograph "Chromatek–Kristall-5000" with a flame ionization detector, manufactured by CJSC SKB "Chromatek". A mixture of methyl esters of 38 fatty acids (F.A.M.E. Mix, C4-C24), 10 mg/ml in methylene chloride was used as a standard. The volume of sample injection with a syringe is 1 μl. The sample is introduced without dilution, after sample preparation. Sample preparation includes distillation of a mixture of hexane and alcohol from a sample of microalgae biomass with a rotary evaporator. The analysis of fatty acids is carried out with the addition of 0.5 ml of methanol solution of potassium hydroxide. Methylation of the sample is carried out within 2 minutes. Then, within 5 minutes, the sample is settled, the resulting solution containing methyl esters is injected into the chromatograph injector with a micro-syringe in a volume of 1 μl. The conditions for analysis by this method are as follows: RESTEK Btx-2330 column (105 m×0.25 mm×0.2 microns); nitrogen carrier gas, gas velocity 20 cm/s; temperature program—8 min at 140°C, 10 min at 250°C; sample input temperature 200°C, hydrogen supply rate– 40 ml/min, nitrogen– 62 ml/min, oxygen– 400 ml/min.

Each experiment in this study was conducted triplicate and presented the value as the mean ± significant difference of three replications.

## Results and discussion

### Growth curves of microalgae cultures

**Fig 1:**
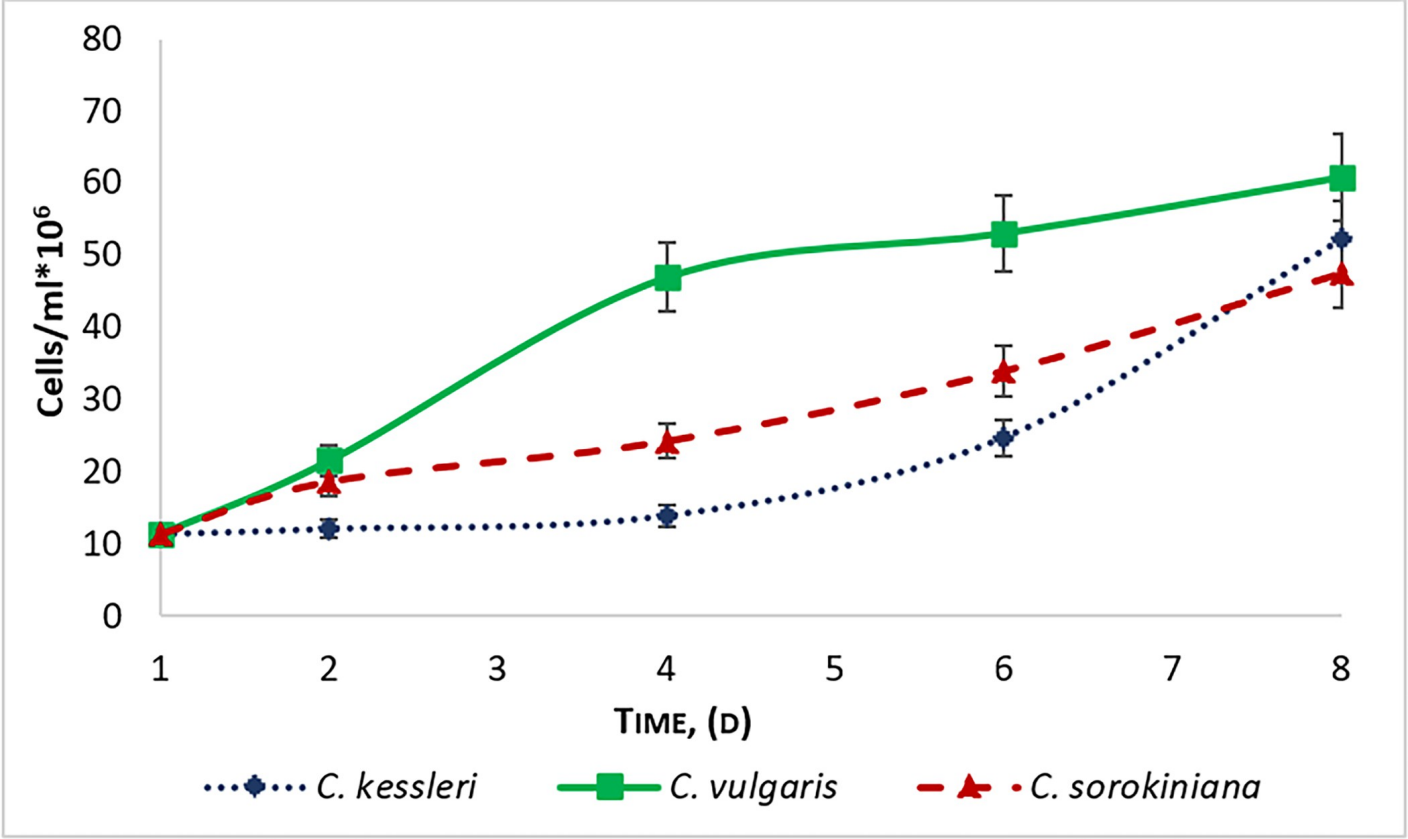
Microalgae growth curves. *С*. *vulgaris* grown with the addition of wastewater; *C*. *sorokiniana* grown on a standard medium; *C*. *kessleri* grown on nutrient medium with nitrogen deficiency.

Fig 1 depicts three growth curves: *C*. *kessleri* grown on nutrient medium with nitrogen deficiency (sample N°2); *С*. *vulgaris* grown with the addition of wastewater (sample N°3) and *C*. *sorokiniana* grown on a standard medium (sample N°4). For the initial growth period and development of microalgae cultures, a lag-phase is often observed which is characterized by the absence of growth or a negative growth rate [32]. At this time, microalgae cells adapt to new environmental conditions [33]. The duration of the period takes from several minutes to several days, depending on the conditions in which the cells were before they were introduced into this medium.

The pH in samples N°2 and N°4 shifted to the alkaline region. In the *C*. *kessleri* sample, the pH rose from the initial 6.8 to 10.3. In the sample of *C*. *sorokiniana*, the pH value at the start of the cultivation was 7.0, and became 9.9 at the end of the experiment.

According to the results, a long lag phase can be observed only in *C*. *kessleri* probably due to the lack of nitrogen in the medium as a long adaptation phase of the microalgae to its new medium. The exponential phase is observed from day 6 to 8. The number of cells of *C*. *kessleri* at the end of cultivation was 52 million cells /ml. A steady and slow increase of microalgae growth can be seen in *C*. *sorokiniana* without reaching the stationary phase. The cell number at the end of cultivation of *C*. *sorokiniana* was about 47 million cells /ml. *С*. *vulgaris* grown with the addition of wastewater shows faster growth compared to the other *Chlorella* samples reaching the highest yield (60 million cells /ml after 8 days of cultivation). The exponential phase is observed from day 1 of cultivation and the exponential phase is reached starting day 4.

### Nutrient removal efficiency by *С*. *vulgaris* (sample N°3)

According to the results, it can be observed that the pH of the medium increased from 7.1 to 10.6. This is often described as being the result of the photosynthetic activity of microalgae under favorable conditions. Combined to the results stated in Fig 1, it could be concluded that the addition of dairy wastewater did not show significant adverse effects on the growth of *С*. *vulgaris*. According to literature, the active growth of photosynthetic microalgae contributes to reducing BOD and coliform bacteria in wastewater [34].

**Fig 2:**
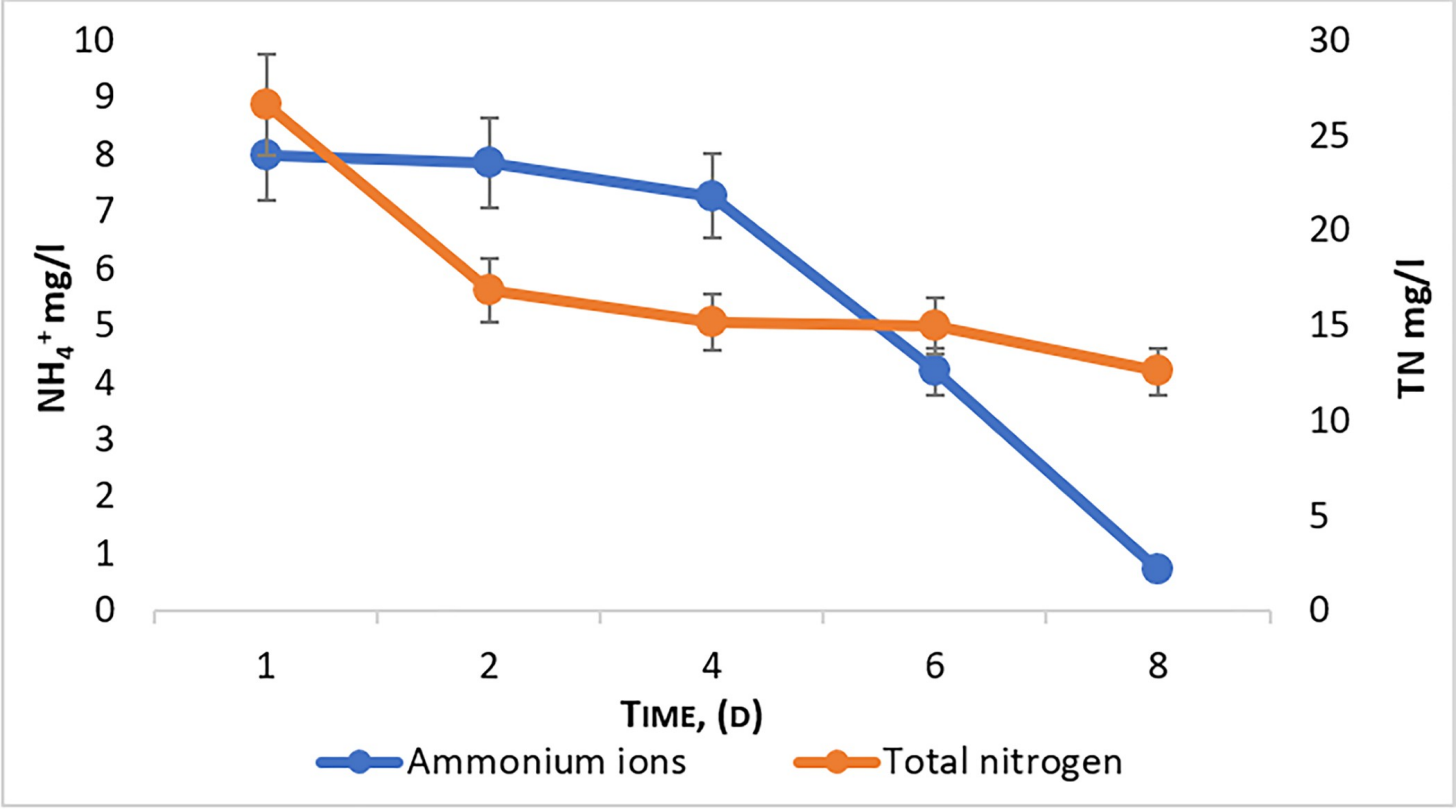
Efficiency of purification from total nitrogen (TN) and ammonium ions (NH4+) during the cultivation of *Chlorella vulgaris*.

The process of transforming ammonium nitrogen from wastewater is called nitrification, when with the help of nitrifying bacteria ammonium nitrogen is oxidized to nitrites, and then nitrites are oxidized to nitrates [35,36]. This process is shown in (Fig 2).

According to the results, the removal efficiency of ammonium nitrogen from wastewater reached 90% and 53% of total nitrogen. This shows that *C*. *vulgaris* could be used as an efficient bioremediator of nitrogen from wastewater.

**Fig 3:**
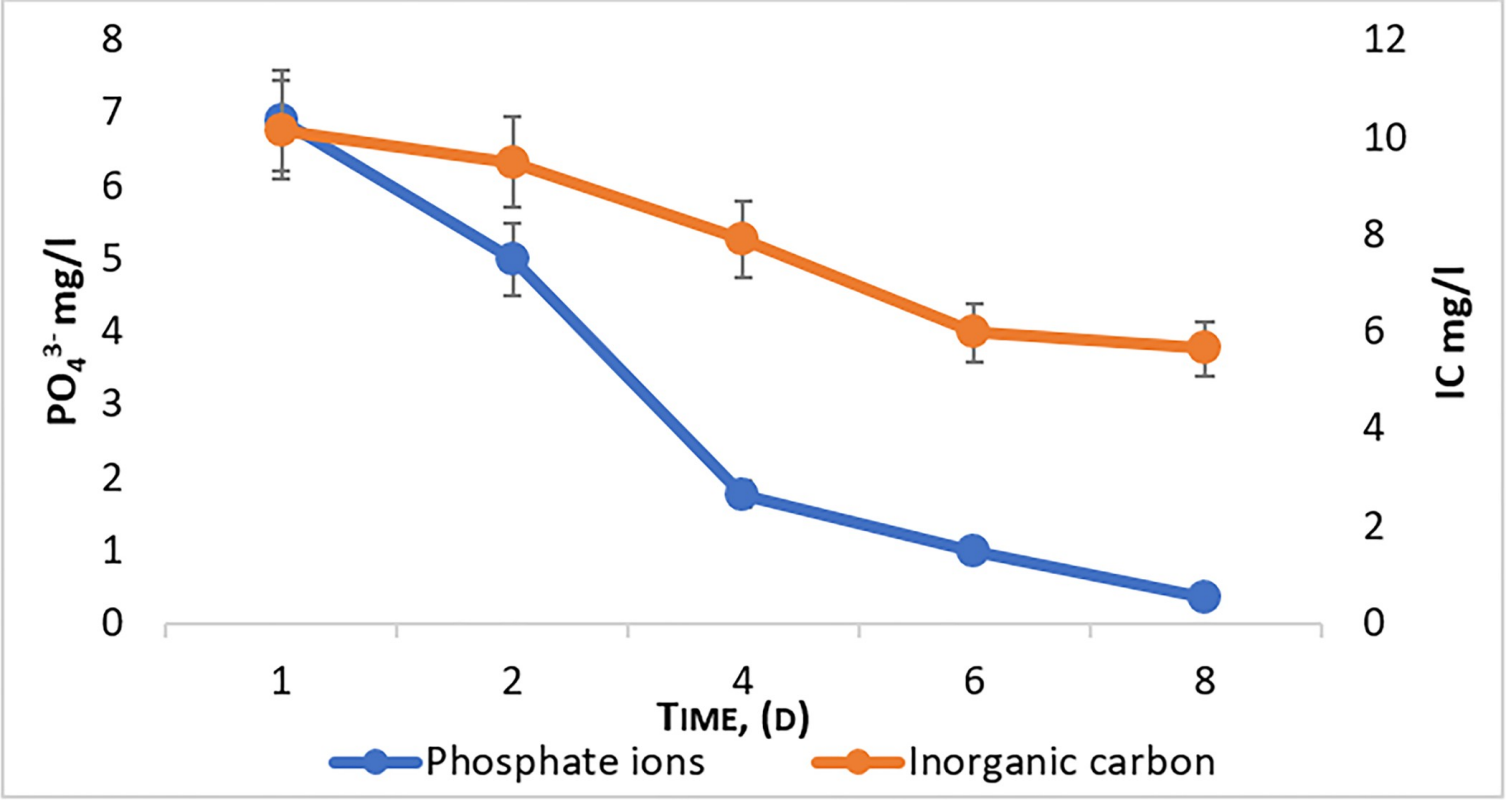
Efficiency of purification from inorganic carbon (IC) and phosphate ions (PO43-) during the cultivation of *Chlorella vulgaris*.

Organic matter is decomposed and used by microalgae biomass as nutrients. During cultivation, microalgae absorb carbon dioxide and release oxygen, which inhibits the decay and fermentation of milk sugar. At the same time, there is a decrease in inorganic carbon and its processing into organic carbon by microalgae [37,38]. In this medium, microalgae grow at a high rate (more than 50 million cells /ml after only 4 days of cultivation) while the decrease in inorganic carbon occurs simultaneously (Fig 3).

The recovery efficiency of phosphate ions reaches 95%. It is known that phosphates are biogenic elements necessary for the growth and development of microalgae [39,40]. So, the cultivation process is characterized by intensive absorption and, consequently, a decrease in the concentration of phosphates in the medium, as well as an increase in the biomass yields. This makes it possible to achieve the main goal of water purification while simultaneously producing biomass.

### Lipid yields and fatty acid composition

**Fig 4:**
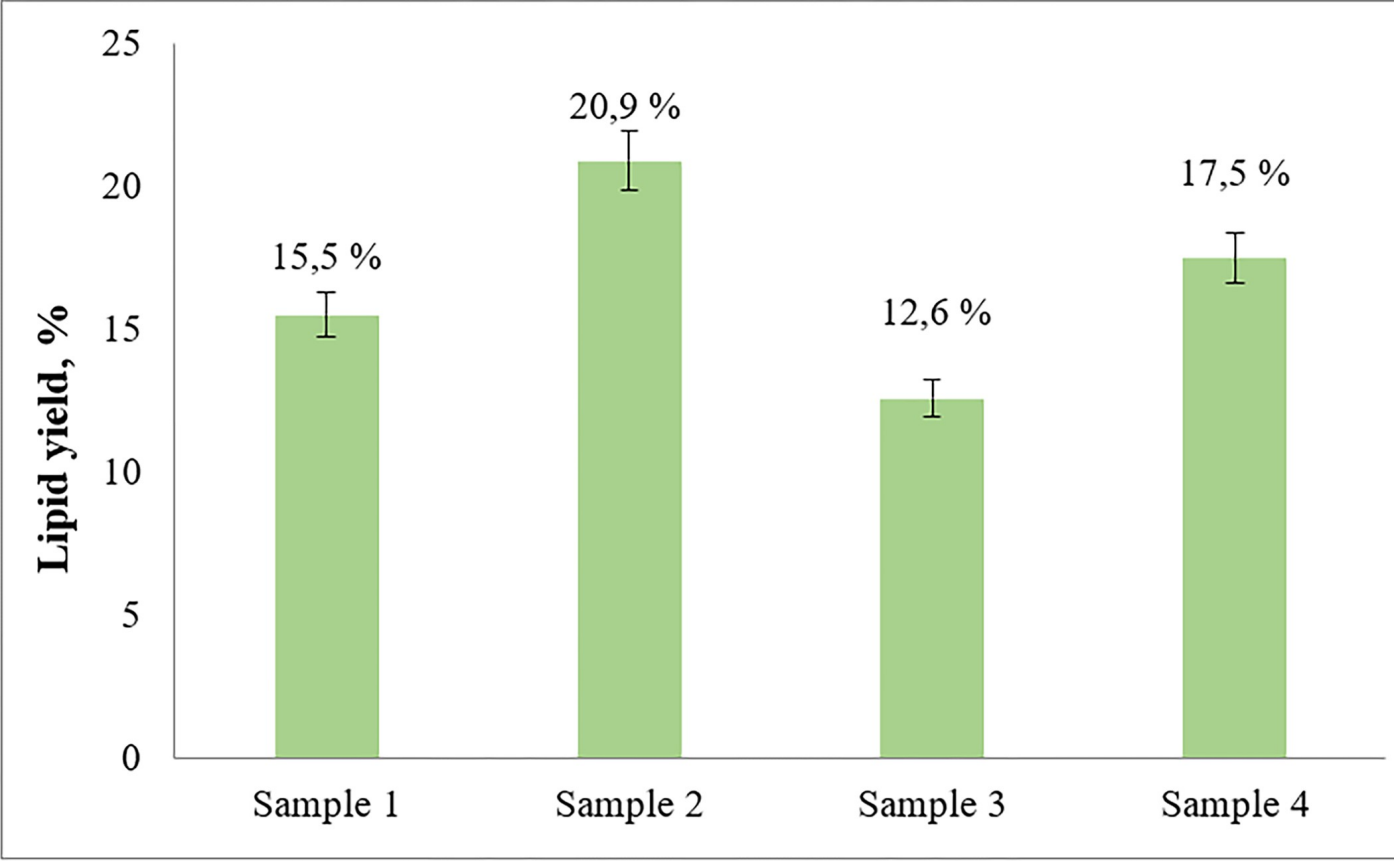
Yield of lipids obtained from various chlorella species. Sample N° 1 –*C*. *kessleri* (SPA "Algobiotechnology"); sample N° 2 –*C*. *kessleri* (laboratory "Industrial ecology" SPbPU); sample N° 3 –*C*. *vulgaris* ("Algotek"); sample N° 4 –*S*. *sorokiniana* (SAG).

Results on the yields of lipids extracted from the dry biomass of the different microalgae species is shown in Fig 4. The highest yield (20.9%) was obtained from the dry biomass of *C*. *kessleri* obtained in laboratory conditions (sample N° 2) followed by *C*. *sorokiniana* (17.5%) (sample N° 4) and *C*. *kessleri* (15.5%) (sample N° 1). The high yield of lipids under nitrogen starvation (sample N° 2) confirms that these conditions do indeed contributes to the accumulation of lipids. However, one of the disadvantages of nitrogen starvation is the low biomass productivity, as demonstrated above in Fig 3, which is consistent with the data found in the literature [41,42]. Nitrogen stress could weaken algal photosynthesis, disrupt the metabolic balance of algal cells, block the transport of energy and nutrients required for proper growth, and gradually stop growth and division [43]. The biomass of *C*. *vulgaris* produced the lowest yield of lipids (12.6%).

The residual moisture content in the dry biomass samples are presented in Table 2. The percentage of moisture content in the biomass was taken into account when calculating the yields of lipids in the dry biomass of microalgae. Results show that the moisture content of all the studied samples is close to 3.5%.

**Table 2:** Residual moisture content in microalgae biomass.

Strain name	Residual moisture content, %
*С*. *vulgaris*	3,50±0,08
*C*. *sorokiniana*	3,11±0,07
*C*. *kessleri grown on nutrient medium with nitrogen deficiency*	3,23±0,08
*C*. *kessleri*	3,33±0,08

**Fig 5:**
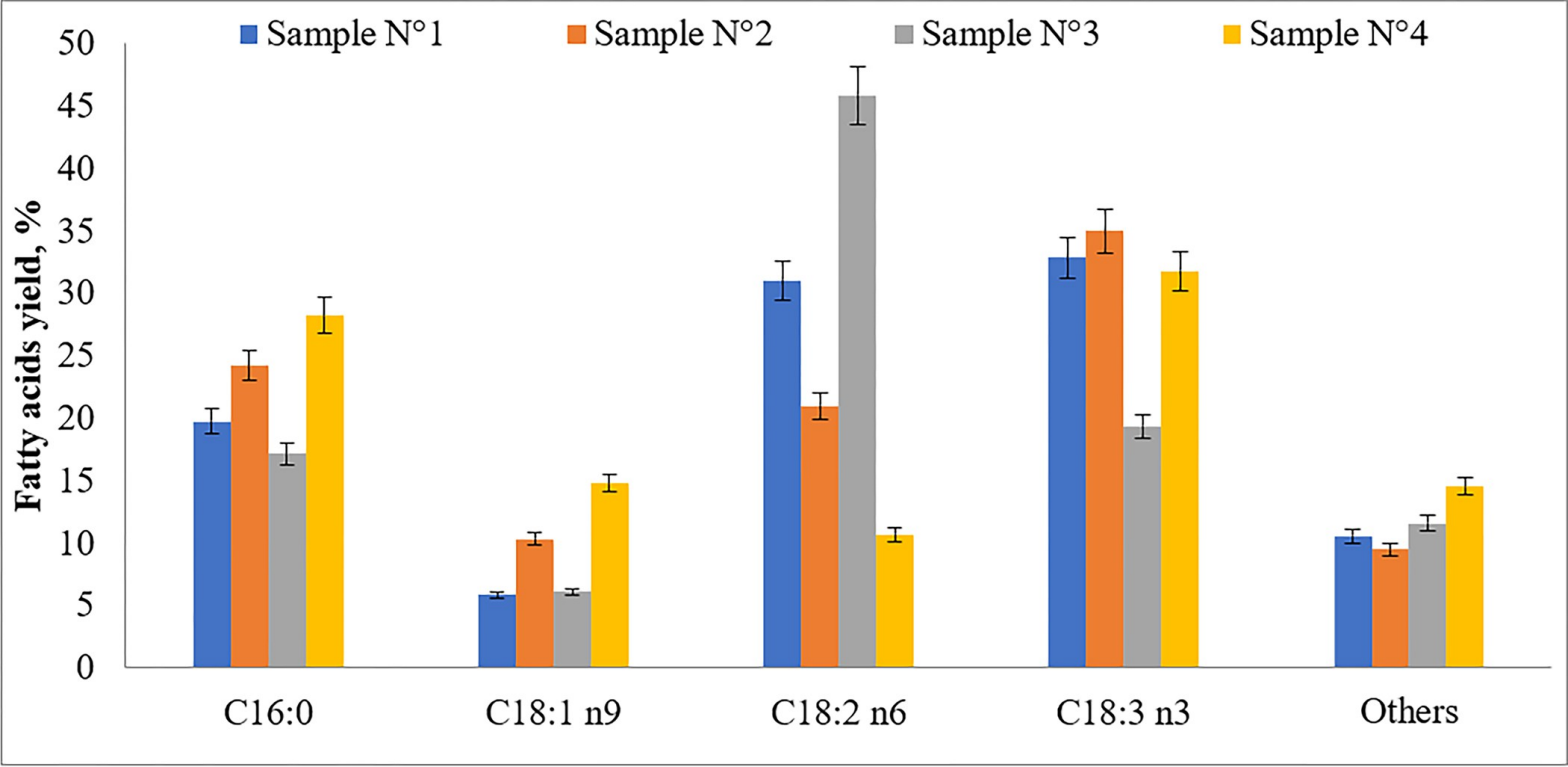
Fatty acid composition of lipids obtained from the dry biomass of various microalgae species. Sample N° 1 –*C*. *kessleri* (SPA "Algobiotechnology"); sample N° 2 –*C*. *kessleri* (SPbPU laboratory); sample N° 3 –*C*. *vulgaris* ("Algotek") sample N° 4 –*C*. *sorokiniana*. Names of fatty acids: Palmitic (C16:0); Oleic (C18:1 n9); Linoleic acid (C18:2 n6); α-Linolenic (C18:3 n3).

The lipid content and their quality must be taken into account when selecting microalgae strains suitable for biofuel production. The presence of specific types fatty acids in sufficient amounds is crucial to ensure the quality of biodiesel. A high concentration of saturated fatty acids gives biofuels good resistance to oxidation and a higher cetane number [44]. A detailed analysis of the fatty acid composition of lipids is presented in Fig 5.

Results show that all four microalgae samples have a similar fatty acid profile. The main saturated fatty acid (SFA) is represented by palmitic acid (C 16:0), which is suitable for the production of biodiesel. The main unsaturated fatty acids (UFA) present in the samples were oleic acid (C18:1 n9); linoleic acid (C18:2 n6) and alpha-linolenic acid (C18:3 n3), which are omega-9, omega-6, omega-3, respectively. In the authors’ study on the cultivation of *C*. *sorokiniana* and *C*. *vulgaris* microalgae in the wastewater of dairy treatment plants, the main acids for both microalgae were C18:3, C16:0, C18:2 [45]. Which is consistent with the results obtained.

In terms of SFAs, which are necessary for the production of biodiesel, the highest percentage was obtained from *C*. *sorokiniana* (37.2%), followed by *C*. *kessleri*, grown in laboratory conditions (29.43%). The lowest concentration of SFA was observed in *C*. *vulgaris* (23.5%). The content of palmitic acid in sample N° 2 is slightly higher than in other samples, which is consistent with the results obtained by the authors [46,47]. Cointet et al. [48] reported that the most abundant saturated fatty acid was palmitic acid, ranging from 32.5 ± 4.3% to 64.6 ± 6.0% for all strains in nitrogen-limited treatment. However, it is also important to mention the need for monounsaturated fatty acids (МUFA) for biofuel production. These fatty acids provide higher fluidity, which is crucial for the quality of biodiesel [49]. Table 3 shows the content of saturated, monounsaturated and polyunsaturated fatty acids in the studied microalgae species. The content of MUFA was the lowest in *C*. *vulgaris* (10.3%) and *C*. *kessleri* obtained from (SPA) "Algobiotechnology" (10.5%). And the highest–in *C*. *sorokiniana* (19.4%) and *C*. *kessleri* grown in laboratory conditions (15.6%).

**Table 3:** The content of SFA, MUFA and PUFA in the studied species of microalgae.

FA, % Microalgae strains	SFA	UFA	
		MUFA	PUFA
*C*. *kessleri*	25,2	10,9	64,3
*C*. *kessleri* (SPbPU lab)	29,4	15,6	54,9
*C*. *vulgaris*	23,5	10,3	66,2
*C*. *sorokiniana*	37,2	66,2	43,1

Polyunsaturated fatty acids (PUFA) are not suitable for the production of biodiesel, but are part of Omega-3. However, these fats are essential for human nutrition, playing beneficial roles in human health [50]. The highest PUFA content was found in sample 1 (*C*. *kessleri*)– 64.3%, followed by *C*. *vulgaris*– 66.1% and *C*. *kessleri* grown in the laboratory—55%. The lowest PUFA content was obtained from *C*. *sorokiniana* (43.4%). Regarding the observed variabilities of the fatty acid profiles, it is known that the chemical composition of microalgae sensibly varies from species to species. However, environmental factors, among which, the composition of the growth medium also play a considerable role in this variability [51]. The accumulation of higher amounts of saturated fatty acids under nitrogen deficiency has been observed when comparing the two samples of *C*. *kessleri*, which confirmed previous results. It has been demonstrated that the expression of elongases and desaturases enzymes in microalgae varies with nitrogen status as a response to stress [52]. *C*. *vulgaris* has accumulated the highest percentage of unsaturated fatty acids compared to other species, which is probably due to the availability of essential nutrients, such as nitrogen and phosphorus, provided in enough amounts in the nutrient medium supplemented with wastewater. Thus, the use of dairy wastewater could be suitable for nutrient remediation but could influence the quality of the obtained biodiesel due to the higher amount of unsaturated fatty acid content. According to the obtained results, the most suitable species among the studied samples for biodiesel production was *C*. *sorokiniana*.

## Conclusions

The performed work shows, that it is possible to carry out a simultaneous process of wastewater treatment and microalgae cultivation. The removal efficiency of 90%, 53% and 95% of ammonium nitrogen, total nitrogen and phosphate ions, respectively, were reached. The efficiency of wastewater treatment from inorganic carbon was 55%, while the maximum growth of biomass was achieved.

The lipid yield and fatty acid profiles provide valuable information about the quality of the biomass as a raw material. According to the conducted analyses, it can be assumed that *C*. *sorokiniana* is the most promising species for biofuel production. The lipid yield was highest in *C*. *kessleri* grown in the laboratory photobioreactor, and occupies the second position in terms of the content of SFA and MUFA and can also potentially be a source of biofuel.
